# Improving the Robustness of Real-Time Myoelectric Pattern Recognition against Arm Position Changes in Transradial Amputees

**DOI:** 10.1155/2017/5090454

**Published:** 2017-04-24

**Authors:** Yanjuan Geng, Oluwarotimi Williams Samuel, Yue Wei, Guanglin Li

**Affiliations:** ^1^CAS Key Laboratory of Human-Machine Intelligence-Synergy Systems, Shenzhen Institutes of Advanced Technology (SIAT), Chinese Academy of Sciences (CAS), Shenzhen 518055, China; ^2^Institute of Biomedical and Health Engineering, SIAT, CAS, Shenzhen 518055, China; ^3^Shenzhen College of Advanced Technology, University of Chinese Academy of Sciences, Shenzhen 518055, China

## Abstract

Previous studies have showed that arm position variations would significantly degrade the classification performance of myoelectric pattern-recognition-based prosthetic control, and the cascade classifier (CC) and multiposition classifier (MPC) have been proposed to minimize such degradation in offline scenarios. However, it remains unknown whether these proposed approaches could also perform well in the clinical use of a multifunctional prosthesis control. In this study, the online effect of arm position variation on motion identification was evaluated by using a motion-test environment (MTE) developed to mimic the real-time control of myoelectric prostheses. The performance of different classifier configurations in reducing the impact of arm position variation was investigated using four real-time metrics based on dataset obtained from transradial amputees. The results of this study showed that, compared to the commonly used motion classification method, the CC and MPC configurations improved the real-time performance across seven classes of movements in five different arm positions (8.7% and 12.7% increments of motion completion rate, resp.). The results also indicated that high offline classification accuracy might not ensure good real-time performance under variable arm positions, which necessitated the investigation of the real-time control performance to gain proper insight on the clinical implementation of EMG-pattern-recognition-based controllers for limb amputees.

## 1. Introduction

Electromyography-pattern-recognition- (EMG-PR-) based approach has great potential to provide intuitive control in myoelectric prostheses with multiple degrees of freedom (DOF), and thus it has been widely investigated in the last two decades [1–14]. However, the existing EMG-PR-based upper limb prostheses are yet to be available for clinical use [15–17], and this could be mainly attributed to the gap between the academics and the industry [7, 8, 18–37]. In order to speed up the clinical implementation of the multifunctional myoelectric prostheses, some disparities between an ideal laboratory setting and practical use of a myoelectric prosthesis, such as the electrode location shift, electrode configuration, muscle contraction variation, muscle fatigue overtime, sampling rate of EMG signals, and the prosthesis weight, were investigated in different research groups worldwide [8, 18–20, 28–32, 38, 39].

Arm position variation is also an important disparity between the practical use of myoelectric prosthesis and laboratory setting. In most previous studies, EMG signals used to train and test a motion classifier were recorded in a specific arm position, thus leading to high classification accuracy in such an ideal experimental setting. In the practical use of myoelectric prosthesis, the arm positions of amputees would unavoidably change while performing several upper limb movements. Hence, when doing a movement in arm positions that are different from the ones used to train a motion classifier, the EMG patterns would be different, and decay in motion classification performance would consequently occur. Recently, a number of research groups have conducted studies with able-bodied subjects and/or arm amputees to evaluate the effect of arm position variation on the classification performance of EMG-PR classifier and proposed several possible approaches to minimize such effect [21–26, 40, 41]. By using offline classification accuracy or error, these studies showed that arm position variation would significantly affect the classification performance of a motion classifier, and they proposed various classification schemes to attenuate the impact of variation in arm position. It is important to note that classification accuracy is the ability of a motion classifier algorithm to identify a desired movement from several classes of movements. A high classification accuracy may be prerequisite for improving the control performance of a multifunctional myoelectric prosthesis, but it might not ensure a good real-time control performance due to the disparities between an ideal laboratory setting and practical use of a myoelectric prosthesis. A previous investigation demonstrated that the offline accuracy may not have a strong correlation with real-time performance of EMG-PR-based prosthesis control [42]. This suggests that using the offline classification performance alone may be insufficient for assessing the usability and clinical viability of EMG-PR-based myoelectric control approaches [42, 43]. Thus it should be required to further examine how variation in arm positions would affect the real-time control performance of EMG-PR and whether the previously proposed approaches [23, 24] are still feasible and robust in reducing the effect of arm position variations in real-time conditions.

In our pilot study conducted with able-bodied subjects, arm position variation was proved to have substantial effect on the real-time motion classification accuracy, and the proposed cascade classifier (CC) method effectively minimized the impact [23]. It was helpful for us to understand the influence of arm position variation on the real-time control performance of EMG-PR and the availability of the proposed CC method in attenuating the influence. However, it remains unclear whether similar impact would be caused by arm position variations on arm amputees who are the final users of a prosthesis. Additionally, it is unknown if the previously proposed CC method and multiposition configuration (MPC) method could provide a robust real-time performance against arm position variations. To provide proper clarification and understanding regarding these issues, five amputees with unilateral transradial amputation were recruited to participate in the current study in which the real-time performance of the CC and MPC as well as the conventional single-position configuration (SPC) method were investigated. In addition, the correlation between offline motion classification accuracy and online performance of EMG-PR control strategy under arm position variations was examined by observing the real-time performance of EMG-PR method. To the best of our knowledge, this would be the first time to investigate these important issues with an attempt to improve the robustness of real-time myoelectric pattern recognition against arm position variations in amputees. The outcomes of this study would provide some useful guides that could aid the development of clinically viable multifunctional myoelectric prostheses.

## 2. Materials and Methods

### 2.1. Participants and Data Acquisition

Five male patients with unilateral transradial amputation (aged from 28 to 47 years) participated in the study. They all have varying degrees of experience in the use of either a cosmetic prosthesis or a myoelectric prosthesis in their daily life. The length of their residual forearms ranged from 5 cm to 25 cm and the demographic information of the subjects is shown in Table 1. All of the subjects gave written informed consent and provided permission for publication of their photographs and data for scientific and educational purposes. And the protocol of this study was approved by the Shenzhen Institutes of Advanced Technology, Chinese Academy of Sciences.

A commercialized biological signal acquisition system called Trigno™ wireless system (Delsys Inc., Boston, USA), which has a base station and 16 wireless hybrid sensors, was used in this study for EMG data acquisition. Each hybrid sensor is composed of a parallel-bar EMG electrode and a built-in triaxial accelerometer electrode. Hence, the EMG signals corresponding to the physiological activities of the arm muscles during contraction and triaxial acceleration signals (ACC) that reflect the arm position variation in 3D space could be acquired simultaneously. The EMG signal resolution is 16 bits and the accelerometer sensitivity is ±1.5 g. Based on the radio frequency (RF) wireless communication of 2.4 G Hz, the hybrid signals could be transmitted to the base station within a range of 40 m. In order to record and process the EMG and ACC signals in real time with MATLAB (version R2010b, the MathWorks, Natick, Massachusetts), a data acquisition card (USB-6218, National Instruments Corp., USA) was used in the current study. The card was connected to the analogue output connector that is localized on the base station of the Trigno wireless system via a DC-A22 unterminated output cable, and its USB port was connected to the desktop. In addition, with the EMGworks Signal Acquisition software equipped with the Trigno wireless system, the EMG signals were filtered with a band-pass filter (20–450 Hz) and ACC signals were filtered with a low-pass filter (50 Hz) and then acquired with a sampling rate of 1000 Hz. Also, power line noise was eliminated from the recorded signals with a 50 Hz notch filter [25].

**Figure 1:**
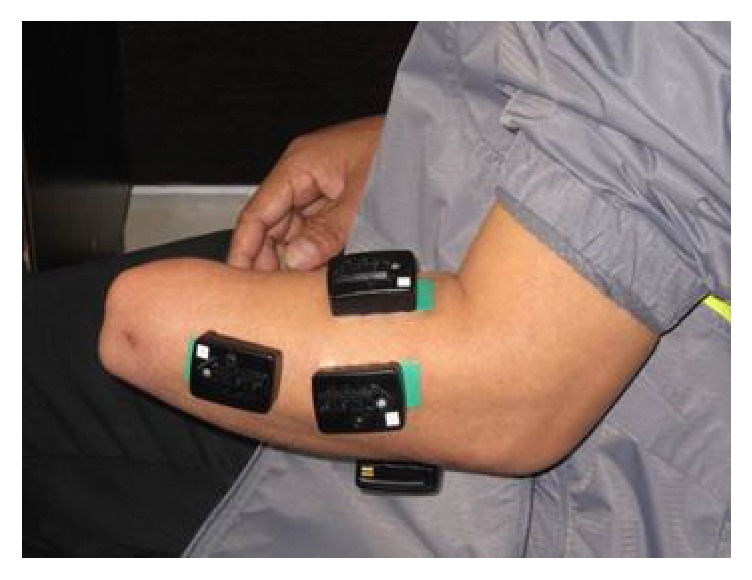
Placement of sensors on residual arm of a transradial amputee.

In the experiment, six hybrid sensors were used to measure the hybrid signals from the residual muscles of the amputated arm. Four of the six sensors were mounted on the remaining arm evenly around the apex of the muscle bulge, 1-2 cm distal to the elbow crease, and another two were placed on the distal end over the flexor muscle and extensor muscle, respectively, as shown in Figure 1. Five arm positions that varied in the sagittal plane and seven classes of forearm movements, which might be commonly involved in daily life activities [23], were considered in the study. The arm positions are as follows:

  P1: straight arm reaching forward (horizontal)
  P2: arm hanging at side, elbow bent at 135°
  P3: arm hanging at side, elbow bent at 90°
  P4: arm hanging at side, elbow bent at 45°
  P5: straight arm hanging at side

The seven forearm movements, including hand open/close (HO/HC), wrist flexion/extension (WF/WE), wrist pronation/supination (WP/WS), and a “rest” class (RT), were considered in the study. With a motion-test environment (MTE) developed using MATLAB programing tool [23], each subject was asked to follow a prompt that displays the arm position images in which the seven forearm movements are performed with a moderate muscle contraction force. Each movement was sustained for 4 seconds and repeated twice, resulting in 8-second hybrid signal recordings (EMG and ACC) per motion class in every arm position. The hybrid signal recordings were then used for classifier training.

### 2.2. Pattern-Recognition-Based Classifier Configurations

To build a pattern-recognition-based classifier, the EMG and ACC signal recordings were segmented into sequential analysis windows with a time length of 150 ms and a time increment of 100 ms (i.e., 50 ms overlapping) [23, 24]. For EMG signal recordings, four commonly used time-domain features [2], mean absolute value (MAV), number of zeros crossings, number of slope sign changes, and waveform length, were extracted from each analysis window and the features from all the analysis windows were concatenated together to form an EMG feature matrix. Similarly, for the ACC recordings, three time-domain features [23, 24], MAV, variance, and maximum value, were extracted from each analysis window and combined together to get an ACC feature matrix. The linear discriminant analysis (LDA) classifier [28, 44] was used as the pattern recognition algorithm because it is much simpler and faster to implement and would not compromise the accuracy of motion classification in comparison to other complex algorithms. In the current study, the first half of the feature matrix was used to train the LDA classifier and the second half was then used to test the trained classifier.

**Figure 2:**
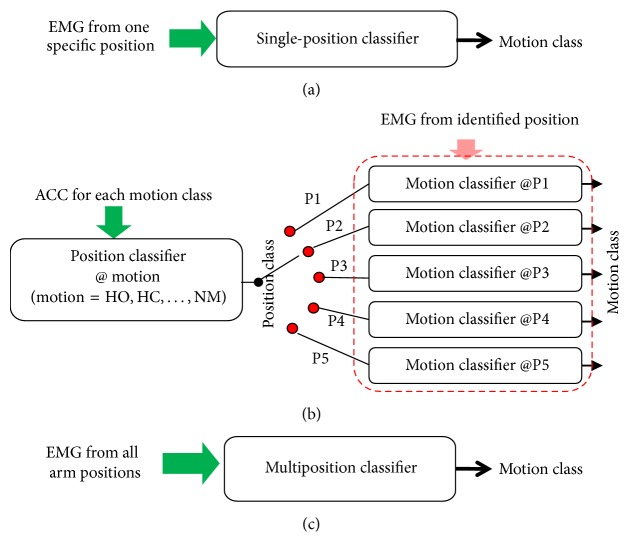
Three classifier configurations. (a) Single-position classifier (SPC). (b) Cascade classifier (CC). CC was composed of seven position classifiers in the first stage and five motion classifiers in the second stage. Each position classifier was trained by ACC signals of each movement, and each motion classifier was trained by EMG recordings from each arm position. In real-time test, the output of the first stage was firstly determined by the most frequently occurring outputs of the seven position classifiers and then used to select a corresponding motion classifier. (c) Multiposition classifier (MPC).

Three classifier configurations including single-position classifier (SPC), two-stage cascade classifier (CC), and multiposition classifier (MPC) were applied in this study and described mathematically by (1)–(3), respectively. The conventional configuration of limb motion classification, that is, SPC, was built to evaluate the real-time effect of arm position variation on myoelectric control. And the SPC was trained with the EMG recordings of all the seven movements in one arm position, as shown in Figure 2(a) and (1). For five arm positions, five single-position classifiers were built, respectively. The CC configuration and MPC configuration were used to attenuate the impact of arm position variation on motion classification performance [23, 24]. As illustrated in Figure 2(b) and (2a) and (2b), the CC configuration consists of two sequential classifiers, in which the first stage that served as a position classifier was trained with ACC for the identification of five arm positions and the second stage that served as a motion classifier was trained with EMG signals for the classification of seven classes of movements.

(1) SPC:  motion_outputm=LDApEMGFeatureMatrixpp=1,2,…,5,

where *p* denotes arm position and *m* denotes the identified motion class.

(2a) CC:  position_outputp=LDAmAccFeatureMatrixmm=HO,HC,WE,WF,WS,WP,NM,

where *m* denotes the motion class. In real-time test, if the *i*th position was identified, then the *i*th motion classifier was selected:

(2b) motion_outputm=LDAiEMGFeatureMatrixi

(3) MPC:  motion_outputm=LDA∑p=15EMGFeatureMatrixp,

where *p* denotes arm position.

A commonly used metric, the classification accuracy which is defined as the number of correctly classified samples over the total number of testing samples, was computed and used to evaluate the offline classification performance of each motion/position classifier [23, 24]. The classification accuracy corresponding to each classifier configuration was defined as follows.

*(1) Classification Accuracy of the SPC*. For each subject, five motion SPCs corresponding to five arm positions were trained using EMG recordings from each arm position, respectively, producing five classification accuracies, among which the maximum value was considered as the offline classification accuracy of the SPC configuration. And the classifier with the maximum classification accuracy was used in real-time experiments.

*(2) Classification Accuracy of the CC*. For each subject, the product of the average accuracy over all the seven position classifiers in the first stage and the average accuracy over all the five motion classifiers in the second stage was considered as the offline classification accuracy of the CC configuration [23, 24]. In the real-time experiments, the arm position was firstly determined by the most frequently occurring outputs of the seven position classifiers and then was used to choose a corresponding motion classifier [23].

*(3) Classification Accuracy of the MPC*. For each subject, there was one MPC classifier for all the positions and motion classes. Thus the classifier was used in real-time experiments and its accuracy in identifying the seven motion classes is the offline classification accuracy of the MPC configuration [24].

### 2.3. Real-Time Myoelectric Classification Experiments and Performance Metrics

**Figure 3:**
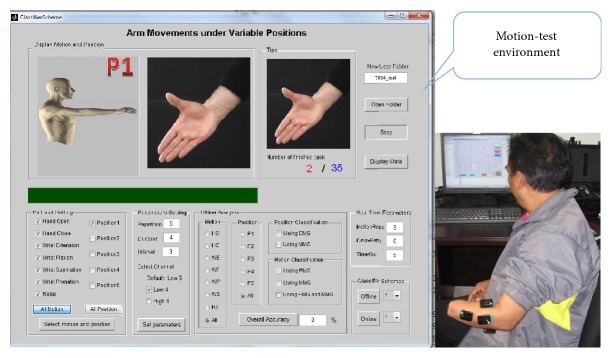
Snapshot of MTE and a representative subject performing the real-time test. The panel of MTE is composed of three main parts, including the parameters selection panel for real-time data recording, parameter selection panel for offline pattern-recognition analysis, and the classifier configuration selection button for real-time test of embedded algorithms.

The real-time myoelectric pattern recognition experiments for prosthetic control were conducted based on MTE. A snapshot of MTE showing a representative subject performing the real-time test is presented in Figure 3.

The three aforementioned classifier configurations were embedded in the developed MTE so as to mimic the real-time control of myoelectric prostheses [23, 24]. For each subject, two real-time experimental sessions were designed. The first is a practical session, in which all the subjects participated in real-time myoelectric motion recognition experiment for more than 30 minutes to get familiar with the MTE. During this session, each of the three classifier configurations was selected and repeated 3–5 times according to the subjects' circumstance. Following the first session, subjects would immediately participate in an experimental session to evaluate the performance of the myoelectric pattern recognition method in real time, and three trials would be performed by using each of the three classifier configurations, respectively. It should be noted that all subjects were blind to the sequence of these classifier configurations before and during the real-time experiment, for the purpose of keeping them from eliciting conscious effort during a specific trial. In each trial, the subjects were instructed to perform each of the seven motion classes for three times in every arm position by following a target movement image and a target arm position image that were randomly displayed on a computer screen. In total, each subject needed to execute 105 movements in a trial. In the real-time experiment, a target movement task was considered as completed if it was successfully performed within a 5-second time limit. Since the current study partly aims to examine the real-time control performance of the three classifier configurations, ten accumulative correct decisions were deemed as a success herein. The real-time recordings including the hybrid signals, real-time instructions, and the real-time prediction results were all stored during the experiment.

**Figure 4:**
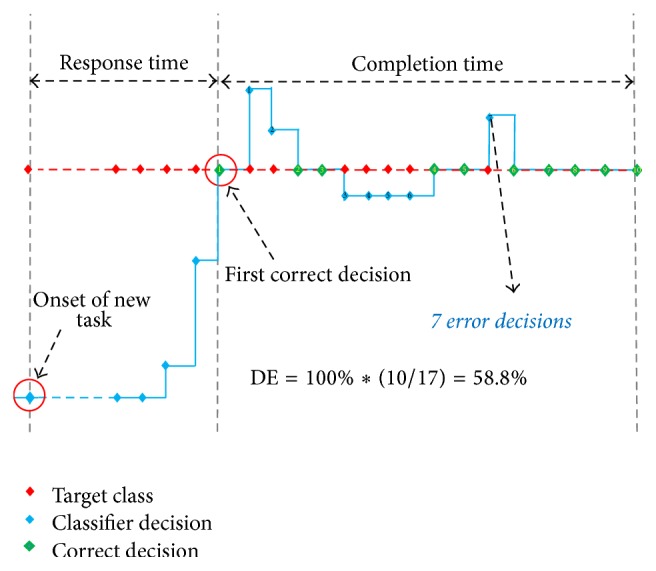
Definition of the four real-time performance metrics. This figure illustrates the process of a successful motion completion in the real-time test, where the red diamonds denote the target motion classes, the blue diamonds denote the identified motion classes, and the green diamonds denote correct identifications when the blue diamonds overlapped with the red diamonds. Ten accumulative correct identifications were deemed as a successful motion completion.

To quantitatively evaluate the real-time performance of the proposed classifier configurations in identifying the seven forearm movements in variable arm positions, four performance metrics were used in the study with three of them shown in Figure 4. Three of the four performance metrics were adopted from previous studies [4, 6, 23, 43, 45], which are response time, motion completion time, and motion completion rate (refer to [4, 43] for the detailed description of these metrics).

Briefly, the response time is defined as the time taken by a subject to correctly perform the target movement for the first time after the onset of a motion task. The completion time was defined as the time from the first correct movement decision to the time the movement was completed. The motion completion rate was the percentage of successfully completed motions out of the total considered motions (105 target motion tasks for each of the three real-time trials) within a given time limit. Another performance metric examined in this study is the dynamic efficiency that was proposed in our previous study [23]. This metric describes how well a target movement was identified in real-time motion classification [23], and it is similar to the real-time accuracy metric employed in Ortiz-Catalan et al.'s study [6]. The dynamic efficiency is defined as the number of correct decisions (target class) over the total number of decisions when successfully completing a movement through the full range of motion. Generally, the longer the completion time is, the lower the dynamic efficiency for a motion task is. Note that the completion time and dynamic efficiency were only calculated for the successful tasks in this study. For each subject, the four real-time performance metrics were computed from the data stored when the subjects performed the real-time experiments based on the MTE system.

### 2.4. Statistical Analysis

A one-way ANOVA test was conducted to assess the statistical difference between the offline and real-time performance of the EMG-PR when using each of the classifier configurations. And, to assess the statistical difference between the different classifier configurations, each of the four real-time performance metrics was used to perform one-way ANOVA. The level of statistical significance was set to *p* < 0.05.

## 3. Results

### 3.1. Offline Classification Accuracy versus Real-Time Completion Rate

Table 2 shows the offline classification accuracy and the real-time completion rate for each subject when using SPC, CC, and MPC, respectively. Compared to the conventional classifier configuration (SPC), the CC achieved higher real-time completion rate and similar offline classification accuracy, while the MPC yielded higher real-time classification performance but lower offline classification accuracy.

The statistical results show that the offline classification accuracy was significantly higher than the real-time motion completion rate when using CC (*p* value < 0.05), but not when using MPC (*p* value = 0.49). These findings suggest that the robustness of the two proposed classifier configurations under variable arm positions and, in addition, a high offline classification accuracy might not ensure a good real-time performance, and some sacrifices of offline classification performance may bring better real-time performance of EMG-PR control approach. The cross-correlation coefficient between the offline classification accuracy and the real-time motion completion rate was also calculated, and 0.09, 0.48, and 0.37 were recorded for SPC, CC, and MPC, respectively. This outcome suggests a weak correlation between the offline and real-time performance metrics.

### 3.2. Overall Real-Time Performance Metrics of Three Classifier Configurations

The average real-time performance metrics across all subjects are summarized in Table 3. The conventional classifier configuration (SPC) had the lowest motion completion rates (64.60%), which are about 8.70% and 12.70% lower when CC and MPC were used, respectively. Although no significant difference was found among the three classifier configurations in terms of motion completion rate (*p* value = 0.52), the increment in motion completion rate (8.70% for CC and 12.70% for MPC) suggests the effectiveness of the CC and MPC in attenuating the impact of arm position variation for the transradial amputees, and the MPC seems better than CC.

Moreover, the other three real-time performance metrics were also analyzed in the study, in which the MPC seems to be the best for its shortest completion time and highest dynamic efficiency.

### 3.3. Motion Completion Rate versus Arm Positions and Motion Classes

**Figure 5:**
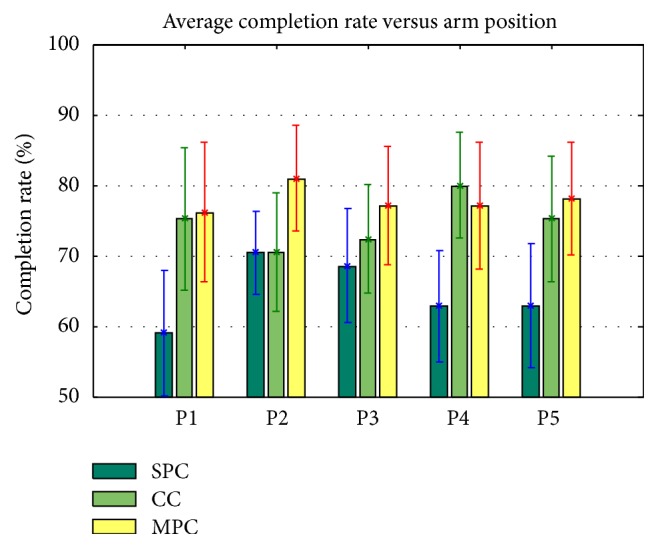
Average motion completion rate versus arm position for all subjects.

Considering that the motion completion rate is a direct measure of the successful rate of a classifier configuration in the real-time condition, the effect of variation in arm position on completion rate was further investigated in the study. The average motion completion rate versus the five arm positions is shown in Figure 5. It is obvious that, among the three examined classifier configurations, the SPC had the worst performance and the MPC had the best performance. The CC had higher value of completion rates in all arm positions in comparison with SPC but lower value of completion rates than MPC except in P4, which may account for the median performance when using CC.

**Figure 6:**
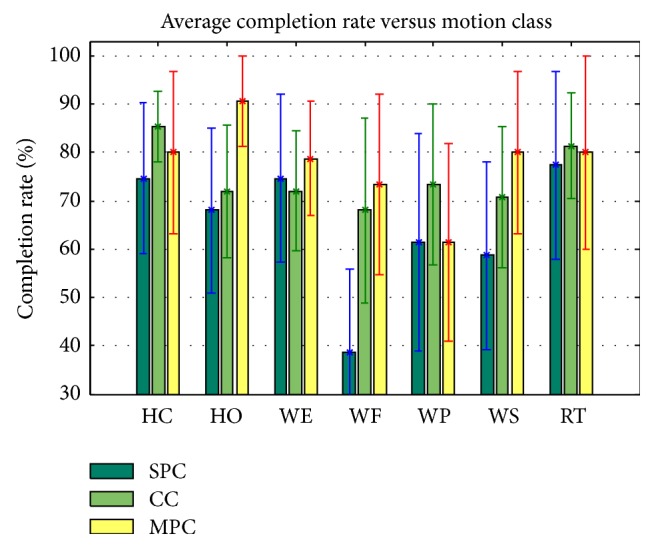
Average motion completion rate versus motion class for all subjects.

Figure 6 illustrates the average motion completion rate versus seven motion classes. Compared to the conventional SPC, the MPC and CC classifiers achieved higher completion rate for almost all the seven classes of movements. And the motion completion rate was relatively stable among the different motion classes when using CC and MPC, while the SPC had a quite low completion rate for WF. In general, the performance of the MPC is slightly better than that of the CC, because, for four of the seven motion classes, the MPC had a higher completion rate than the CC and, for other three motion classes, the MPC got a lower or similar completion rate in comparison to the CC.

## 4. Discussion

To facilitate the practical application of EMG-PR-based prosthesis, the impact of arm position variation on the real-time performance of myoelectric control and the robustness of two previously proposed solutions [24] against variable arm positions were assessed in the current study. To make the investigation more clinically relevant, five transradial amputees whom we assumed to be the final user of upper limb prostheses were recruited to participate in this study. Additionally, the correlation between the offline classification accuracy and the real-time motion classification performance under variable arm positions was analyzed. Compared with previous studies that were conducted on normally limbed subjects and/or arm amputees by means of postprocessing (offline) analysis [21, 22, 24, 26], this study represents a further step towards the practical use of EMG-PR-based method in multifunctional myoelectric prostheses in clinical settings. The outcomes obtained from the real-time experiment with the transradial amputees would provide some useful insights for the design of EMG-PR-based controllers.

In the absence of commercially available multifunctional prosthesis systems with multiple DOF, a custom-made MTE system was developed to mimic the practical control of a physical prosthesis in the study. Based on the MTE system, the impact of arm position variation on real-time myoelectric control and the usability of the two previously proposed classifier configurations were assessed by embedding the corresponding classifier configurations into the MTE system. Four real-time performance metrics including the motion completion rate, response time, completion time, and the dynamic efficiency were used in the study to quantitatively evaluate the real-time performance of each proposed classifier strategy. Note the definition of successful tasks in the current study was consistent with that introduced in some previous studies [4, 43]. A motion task was considered successful if it was correctly identified with ten accumulative decisions within a designated time limit of 5 s. This time limit was chosen based on the practical experience of the subject. The users of prostheses may be frustrated and may not want to continuously perform a movement since the prosthetic operation would be too slow after approximately 5 s attempting to perform a movement [4, 43].

The offline motion classification accuracy and the real-time motion completion rate are both important to assess the performance of EMG-PR approach, and the relationship between them was firstly investigated in this study. A weak correlation independent of the classifier configuration was observed. This finding is consistent with the conclusion of a previous study that did not consider the effect of variations in arm position on limb movement classification [42]. In addition, the two proposed classifier configurations (CC and MPC) both yielded higher real-time completion rates in comparison to the SPC, but contrary to the variation tendency of the offline classification accuracy. This outcome conflicts with the empirical understanding (i.e., degradation in offline classification accuracy may decay the real-time motion classification accuracy of myoelectric control). It suggests that the classification accuracy calculated by postprocessing EMG recordings may not be a direct measure of real-time performance. More specifically, the high offline motion classification accuracy maybe a necessary but not a sufficient condition to ensure a good real-time control performance of multifunctional myoelectric prostheses. In the current study, TR05 is a good case in point, for whom the offline classification accuracy was acceptable (81.60–91.90%), but real-time motion completion rate was quite low (41.90–49.50%). The evident decrease in motion completion rate for TR05 may be mainly attributed to his short residual forearm (only with a length of 5 cm), because the subject could not feel the contraction of the remaining forearm muscles very well and thus felt a bit irritated to elicit repeatable muscle contractions in variable arm positions during the experiment. Moreover, other factors such as muscle contraction force, mild muscle fatigue, and change in skin independence might also influence the real-time performance of myoelectric control. Therefore, the investigation of a real-time control performance is necessary to gain proper insight on the feasibility of clinically implementing EMG-PR-based controllers for upper limb amputees.

In comparison to the conventional SPC configuration, the increment in motion completion rate when using CC and MPC (Tables 2 and 3) indicates that the two proposed solutions would effectively reduce the impact of arm position changes in real-time pattern-recognition control of a multifunctional myoelectric prosthesis. Furthermore, the MPC configuration has a higher motion completion rate in comparison to the CC configuration. The increased generalization of the MPC configuration caused by combining the training set of EMG data from all the arm positions might account for its high performance in real time compared to the other two configurations. The variation of the motion completion rate with respect to the different arm positions and motion classes for the three classifier configurations was also investigated (Figures 5 and 6). The results show that the MPC classifier configuration did not work well in all the arm positions and arm movements, but it overall outperformed the CC configuration. However, in our previous study the CC classifier achieved a higher offline classification accuracy than the MPC classifier [24]. The inconsistent findings between this online study and the previous offline analysis again suggest that the control issues of a prosthesis associated with its practical applications should be validated in a real-time condition before it could be clinically viable.

In addition to the motion completion rate, other three real-time performance metrics were also analyzed in the study. Note that the completion time and dynamic efficiency were used to measure how well a motion task could be finished, and they were only calculated for successful tasks. And the response time reflects the reaction speed of subjects. In the current study, there was no much difference among the three real-time performance metrics when examined on the three different classifier configurations (CC, MPC, and SPC). The results show that the two proposed classifier configurations (MPC and CC) have somewhat similar response time in comparison to the SPC configuration. However, the CC method has a relatively lower response rate compared to MPC and SPC.

The limitation of the current study is that the arm positions and motion classes were randomly presented to the subject in the real-time experiment. Functional tasks such as picking objects from a table and putting them in a box and drinking a cup of water were not included in the movement design of the current study. Such tasks usually involve multiple degrees of freedom and are more close to real life forearm movements. Hence, in our future work, a number of functional tasks would be considered when designing the forearm movements.

## Figures and Tables

**Table 1:** Demographic information of the transradial subjects.

Subjects	Age	Residual limb	Amputated since	Type of Prosthesis	Residual forearm length (cm)
TR01	28	Right	>11 years	Myoelectric	18
TR02	47	Left	>16 years	Cosmetic	25
TR03	29	Right	>10 years	Cosmetic	23.8
TR04	44	Left	>13 years	Myoelectric	25
TR05	30	Right	>14 years	Myoelectric	5

Note the length of residual forearm was measured from the elbow joint.

**Table 2:** Offline motion classification accuracy and real-time motion completion rate for each amputee when using SPC, CC, and MPC, respectively.

	Classification accuracy (%)			Completion rate (%)		
	SPC	CC	MPC	SPC	CC	MPC
TR01	95.20	95.10	93.80	82.9	92.40	97.10
TR02	95.90	95.80	85.90	57.1	78.10	76.20
TR03	92.60	92.50	82.60	62.9	75.20	75.20
TR04	90.70	90.60	75.00	78.1	78.10	88.60
TR05	91.90	91.80	80.60	41.9	42.90	49.50
AVE + STD	93.30 ± 2.20	93.20 ± 2.20	83.60 ± 7.00	64.60 ± 16.50	73.30 ± 18.30	77.30 ± 18.00

**Table 3:** Average real-time performance metrics across all amputees when using SPC, CC, and MPC, respectively.

Classifier Configuration	Completion Rate (%)	Response Time (s)	Completion Time (s)	Dynamic Efficiency (%)
SPC	64.6 ± 16.5	1.12 ± 0.17	1.29 ± 0.14	82.60 ± 6.10
CC	73.3 ± 18.1	1.08 ± 0.10	1.43 ± 0.24	78.60 ± 8.50
MPC	77.3 ± 17.9	1.22 ± 0.15	1.24 ± 0.17	84.70 ± 6.80
